# Effects of standardized language on remote ultrasound-guided percutaneous nephrolithotomy training: A mixed-methods explorative pilot study

**DOI:** 10.1016/j.heliyon.2023.e19629

**Published:** 2023-08-30

**Authors:** David Denisov, Coral Castro-Olmo, Leslie Bernal Charondo, Heiko Yang, Sandrijn Van Schaik, David Bayne

**Affiliations:** a School of Medicine, University of California San Francisco, San Francisco, CA, USA; b School of Medicine, Universidad Central del Caribe, Bayamón, PR, Puerto Rico; c Department of Urology, University of California San Francisco, San Francisco, CA, USA; d Department of Pediatrics, University of California San Francisco, San Francisco, CA, USA

**Keywords:** Surgical training, Simulation training, Telementoring, Distance learning, Communication, Surgical models

## Abstract

**Background:**

Remote teaching of procedural skills has demonstrated equivalence in knowledge acquisition compared to in-person teaching. Variations in terminology for probe and needle movements may serve as a barrier in remote training of ultrasound (US)-guided renal access for percutaneous nephrolithotomy (PCNL). This pilot study investigated the utility of standardized terminology in remote training of US-guided renal access for PCNL.

**Hypothesis:**

Standardization of verbal terminology to describe US probe and needle movement instruction improves remote teaching of US-guided renal access.

**Methods:**

Fifteen urology residents (PGY1-6) were stratified by year and randomized into two groups. We provided participants with images illustrating US probe and needle movements labeled with predetermined standardized terminology for the intervention group and images without labels for the control group. Both groups were asked to perform US-guided renal access on a training mannequin with a remote faculty educator with (intervention) or without (control) use of standardized movement instructions. Quantitative outcomes included number of attempts and time to achieve access. All trainees completed pre- and post-session surveys and participated in focus groups; authors conducted thematic analysis of focus group transcripts.

**Results:**

Differences in primary outcomes between groups, including number of attempts and time to achieve access of the renal pole, were not statistically significant. Analysis of focus group interviews revealed that the use of standardized terminology in the setting of remote training can reduce trainee confusion by clarifying ambiguity in educator feedback.

**Discussion:**

Use of standardized terminology during remote surgical skills training allows for more effective feedback to trainees.

## 1 Background

Interactive remote teaching in the form of webinars and virtual simulation sessions is an effective educational strategy to teach urology residents procedures and has comparable knowledge acquisition to in-person teaching [[1], [2], [3]]. Remote teaching is an attractive alternative to in-person teaching for procedural skills as it allows for immediate feedback during simulation-based medical education, offers the opportunity to record footage for distribution among peers and faculty, and provides a flexible learning pace [ [[4], [5], [6]]]. Additional advantages include a reduction in travel-related expenditures and flexible scheduling [[3], [4], [5]]. The COVID-19 pandemic, as well as obstacles to delivering in-person training to remote areas of the world further highlight the importance of virtual alternatives to in-person teaching of surgical skills. However, the remote approach to teaching procedural skills has limitations. For example, in person, instructors are unable to physically correct the hand movements of trainees. Due to this limitation, effective verbal communication is critical.

Several reports have addressed variations in terminology used to describe device manipulation, particularly ultrasound (US) probe movement [[7], [8], [9]]. However, to our knowledge, the potential benefits of standardizing of terminology in procedural settings have not been studied. Understanding how standardized terminology impacts remote learning can facilitate the design of effective remote education curriculums.

Variations in US probe movement terminology may be particularly problematic for remote teaching of urologic procedural skills. US is a vital tool in the management of a wide variety of urological conditions, including surveillance and surgical treatment of kidney stones. Percutaneous nephrolithotomy (PCNL) is a procedure that relies heavily on accurate needle placement guided by fluoroscopy and/or US. However, there is a learning curve for achieving proficiency in US-guided access[10,11]. This explorative pilot investigates the utility of standardized terminology in the context of remote training of US-guided access percutaneous nephrolithotomy.

## 2 Methods

### 2.1 Setting and participants

We recruited a convenience sample of fifteen urology residents post-graduate year (PGY) 1–6 in May 2022 and randomized them to an intervention (n = 7) and control (n = 6) group. The simulation was performed using ultrasound machines and a Blue Phantom Renal Biopsy Ultrasound Training Model Mannequin [12].

### 2.2 Intervention

**Fig. 1:**
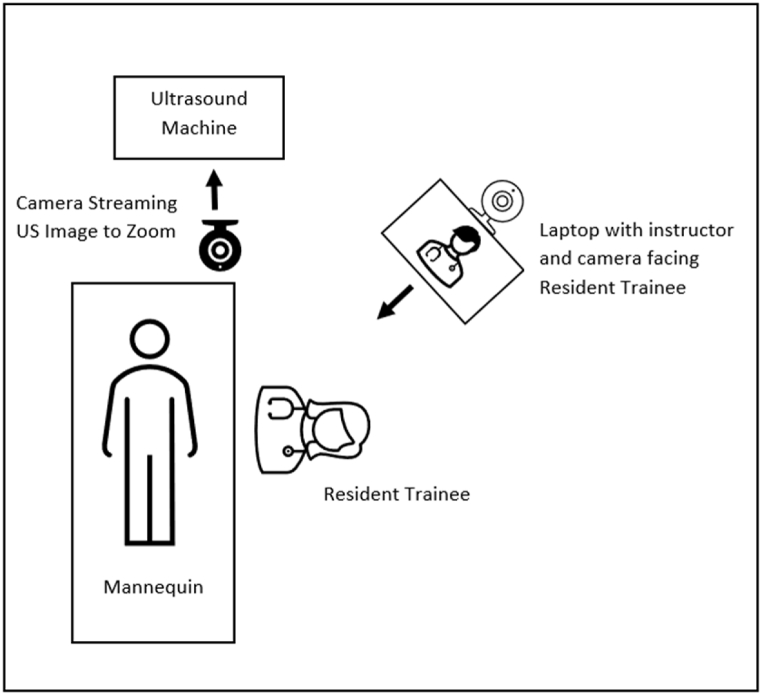
Two laptop camera training set-up. Remote instructors view US image and guide the trainee via Zoom.

The two groups were separated into different rooms. We provided the intervention group with printed learning material containing visual representations of procedural movements and related standardized terminology. The control group was provided with the same images, but without terminology to describe the movements depicted. Residents in both groups practiced renal ultrasound imaging on each other while waiting for individual training sessions. Individual training sessions were conducted in a private room. Participants were asked to achieve renal pole needle access with US guidance using an 18G echo tip Chiba needle. Two faculty instructors in a separate room served as educators from a remote location via video conference and provided feedback about the trainees' needle and probe movements (Fig. 1). Each instructor guided participants from both groups, alternating every two trainees. Educators provided instruction using the standardized terminology for intervention group participants and nonspecific, undefined language for control group participants (e.g., “up” vs “caudal”). Individualized sessions lasted 5 min each.

### 2.3 Outcomes

We collected quantitative and qualitative data to examine the impact of our intervention. We measured number of needle passage attempts and time to achieve renal access. We designed a pre-training survey to assess levels of comfort with US-guided PCNL (Supplemental Material 1) and a post-training survey to measure satisfaction with the remote training session (Supplemental Material 2).

To further explore residents' perceptions of the remote teaching sessions, we developed a semi-structured focus group guide with questions about residents’ general impressions about the session and perceived helpfulness of learning materials (Supplementary Material 3). Two authors conducted in-person, semi-structured focus group interviews. We conducted separate focus groups for the intervention and control participants to avoid discussion between the groups and unintentional revelation of the intervention.

Both focus groups were audio-recorded and transcribed using artificial intelligence software (Otter.ai, Los Altos, CA); two authors reviewed both transcripts, edited them for accuracy, and removed identifying information before analysis. The control focus group lasted approximately 10 min, and the intervention focus group lasted approximately 14 min.

### 2.4 Analysis

For our quantitative data, we calculated descriptive statistics and performed student's t-tests to examine differences in outcomes between groups. Statistics calculated included mean, standard deviation (SD), range, and p-value (Table 1). The first two participants were asked to obtain upper pole access but were unable to obtain access in the time allotted and were not included in the quantitative analysis.

**Table 1:** Quantitative outcomes.

Outcomes	Intervention (n = 7)	Control (n = 6)	P value
Time to access lower pole target in seconds: mean (SD), range	170 (73), 58–278	204 (93), 50–300	0.26
False passages of lower pole target: mean (SD), range	2 (0.58), 1–3	2 (0.82), 1–3	1.0
Satisfaction with training session: mean (SD), range	4.57 (0.79), 3–5	4.67 (0.52), 1–5	0.805
Satisfaction with training materials: mean (SD), range	3 (1.29), 1–5	3.5 (1.05), 2–5	0.485

n = Number of students per group. SD = Standard Deviation. *P*-values were determined by unpaired Student's t-test.

For our qualitative data, we analyzed focus group interview transcripts using thematic analysis. The authors reviewed both transcripts independently to generate potential coding categories and met to discuss findings to generate a preliminary codebook. The authors coded each transcript independently using this codebook, reconciled discrepancies, and further refined the codebook. One author coded each transcript independently using this final codebook and reconciled discrepancies (Supplemental Content 4). All authors reviewed coded excerpts to identify larger themes through iterative discussions and organized codes using Dedoose analytic software (Version 8.3.35, Los Angeles, CA: SocioCultural Research Consultants, LLC).

## 3 Results

### 3.1 Quantitative outcomes

All fifteen trainees’ responses were included in the survey and focus group analyses. Pre-survey results demonstrated a wide range of experience and comfort with PCNL (Table 2). Mean time to access (170 s vs 204 s, P = 0.26) and the mean number of attempts to access (2 vs 2, p = 1.0) were not statistically significant between groups (Table 1). There were no statistically significant differences between groups in ratings measuring satisfaction with the session (p = 0.805) or training materials (p = 0.485) (Table 1).

**Table 2:** Pre-survey results.

Number of Trainees that previously participated in US-Guided PCNL	13	
Median (IQR) number of previous US-guided PCNL experiences	10 (0–40)	
Roles Performed during Previous US-Guided PCNL	Observing	13
	Manipulating Probe	11
	Needle Insertion	9
Comfort Obtaining US-guided PCNL access	None of the Cases	4
	Some Cases	4
	About half of the Cases	3
	Most Cases	3

IQR = interquartile range.

### 3.2 Qualitative outcomes

Through our qualitative analysis we identified two major themes from our focus groups:

(1) Standardized terminology clarifies ambiguity.
(2) Although thought overall to be beneficial, Junior trainees found less benefit to standardized terminology relative to senior trainees.

#### 3.2.1 Standardized terminology clarifies ambiguity

Regardless of the group, trainees appreciated real-time and specific feedback from remote faculty. Intervention group trainees appreciated the incorporation of specific terminology in the feedback received on their US probe and needle movements. Intervention group trainees also used standardized terminology when seeking guidance or clarification from educators.

“I liked the feedback from [the faculty] … [move] the probe in the right direction to touch that, move cranial … I think that at least for our high level of training, it's able to translate well. I can see as … a first year or second year not having someone standing next to me {could be} difficult.”

Control group trainees were confused by ambiguous terminology due to a lack of specific definitions of the terms.

“One time … I was directed to place [the needle] below the probe but … be more specific about what is below.”

#### 3.2.2 Junior trainees found less benefit from standardized terminology

Standardized terminology relating to US probe and needle movements resulted in reduced benefit for junior residents with limited prior exposure to the procedure and renal anatomy.

“There's a very wide range of experience levels in the group … the instructions need to be tailored to each person. Some of us have been doing [US-guided PCNLs] for years and some have never done kidney ultrasound.”

#### 3.2.3 Suggested areas of improvement

Trainees also suggested areas for improvement. Intervention group participants requested having a way to specifically label or point out anatomy on the renal ultrasound image. This demonstrated an unexpected ambiguity in communication that would be difficult to address even with standardized language. This problem could be addressed via the use of screen sharing and annotation tools.

“It was … really hard for us to know which [calyx they were] referring to … In person, you can point to this calyx. But if there was a way for the remote person to be able to make notes on the screen that you're looking at.”

## 4 Discussion

This explorative pilot study examined the impact of standardized language on learning of ultrasound-guided percutaneous nephrolithotomy through remote training. While the quantitative data collected as part of our study lacked statistically significant outcomes, the qualitative data provided valuable insight from trainees. Trainees perceived the use of standardized terminology during remote surgical skills training to be a more effective method for communicating instructions for complex surgical tasks. This was particularly true for senior trainees with increased prior exposure to the standardized terminology utilized. Therefore, early exposure to standardized terminology should be encouraged.

Standardized terminology can be integrated into all procedural and surgical curricula to promote familiarization, such as didactic courses and simulation labs [13]. Variations and ambiguity in terminology exist among movements associated with other procedures, such as laparoscopy.^15^ Achieving consensus among stakeholders regarding the specific terminology used for a procedure allows for reduced confusion and enables future research[14,15]. Trainee comprehension of standardized terminology can be assessed alongside technical skills via simulation-based activities [16,17]. Surgical educators may also benefit from faculty development that integrates reconciliation of previously learned lexicon[18].

The benefits of standardized terminology are not limited to the simulation setting. For example, ambiguous language in the operating room is common and has been associated with near misses [19,20]. Thus, the use of standardized language in the operating room can promote positive patient outcomes.

Our study has some important limitations, including a small sample size and the short duration of individual training sessions. This small sample size may have resulted in insufficient power to detect any difference in quantitative data. The short duration of individual training sessions prevented collection of more longitudinal data, for example, time to access middle and upper poles. Nonetheless, we believe this study offers an initial exploration of the utility of standardized language in US-guided training and provides useful information that can guide future studies with greater sample sizes and more extensive training formats.

## 5 Conclusion

This study was created with the ultimate goal of implementation in a global health curriculum (i.e., to effectively teach residents at distant sites). Future studies should test a similar protocol in a global health procedural education setting and confirm the impact on actual learning outcomes with a larger sample size. Further, it would be useful to explore this topic with different surgical skills where variations in procedural terminology exist.

## Ethics statement

This study was reviewed and approved by UCSF Human Research Protection Program (HRPP) - Institutional Review Board (IRB), with the approval reference IRB #21–33915. This study complies with all required regulations. All participants provided informed consent to participate in the study.

## Author contribution statement

David Denisov - conceived and designed the experiments; analyzed and interpreted the data; wrote the paper.

Coral Castro-Olmo - analyzed and interpreted the data;

Leslie Bernal Charondo - conceived and designed the experiments; performed the experiments; analyzed and interpreted the data; contributed reagents, materials, analysis tools or data; wrote the paper.

Heiko Yang - performed the experiments; wrote the paper.

Sandrijn Van Schaik - analyzed and interpreted the data; wrote the paper.

David Bayne - conceived and designed the experiments; performed the experiments; contributed reagents, materials, analysis tools or data; wrote the paper.

## Data availability statement

Data will be made available on request.

## Declaration of competing interest

The authors declare the following financial interests/personal relationships which may be considered as potential competing interests:David Bayne reports financial support was provided by National Institute of Diabetes and Digestive and Kidney Disorders.
