# Supplementary material for: Effects of standardized language on remote ultrasound-guided percutaneous nephrolithotomy training: A mixed-methods explorative pilot study
Source: Heliyon. 2023 Aug 30;9(9):e19629. doi: 10.1016/j.heliyon.2023.e19629 (PMC10558858; doi:10.1016/j.heliyon.2023.e19629)
Supplement: Multimedia component 1 [file mmc1.pdf]

Default Question Block

Name

Year

PGY1

PGY2

PGY3

PGY4

PGY5

PGY6

Zoom Teaching Session

	1 (Not at all)	2	3 (Neutral)	4	5 (Extremely)
I found this zoom training valuable overall.	○	○	○	○	○
I found the pre-session learning materials helpful.	○	○	○	○	○
I feel more prepared to assist in an ultrasound guided PCNL.	○	○	○	○	○
I feel more prepared to perform an ultrasound guided PCNL.	○	○	○	○	○

What did you find most valuable about this training?

What would make this training more valuable?

Is there anything else you'd like to share with us?

Powered by Qualtrics
