# Supplementary material for: Effects of standardized language on remote ultrasound-guided percutaneous nephrolithotomy training: A mixed-methods explorative pilot study
Source: Heliyon. 2023 Aug 30;9(9):e19629. doi: 10.1016/j.heliyon.2023.e19629 (PMC10558858; doi:10.1016/j.heliyon.2023.e19629)
Supplement: Multimedia component 2 [file mmc2.pdf]

Default Question Block

We are collecting data to study the effectiveness of this course. Survey/interview responses will be kept confidential. If you consent to participate in the study, please click "yes" below and proceed to the survey. Your participation will not affect your education or future status at [redacted]

If you have questions about the survey or the study, please contact [redacted] at [redacted] If you have questions or concerns about your rights as a research participant, you can call the [redacted] Institutional Review Board at [redacted] Thank you!

Yes

No

Name

Year

PGY1

PGY2

PGY3

PGY4

PGY5

PGY6

Have you previously participated in percutaneous nephrolithotomy (PCNL) cases?

Yes

No

Approximately how many PCNL cases have you participated in?

Have you previously participated in ultrasound guided PCNL cases?

Yes

No

Approximately how many ultrasound guided PCNL cases have you participated in?

What roles have you had during ultrasound guided PCNL?

Observing

Manipulating the Probe

Inserting the needle

Are you comfortable obtaining ultrasound guided percutaneous access on a patient?

	None of the Cases	Some Cases	About Half of the Cases	Most Cases	All Cases
I feel confident achieving ultrasound guided access.	○	○	○	○	○

Is there anything you would like to share with us?

Powered by Qualtrics
